# A Successful Cesarean Section in Country Practice

**Published:** 1906-01

**Authors:** S. A. Reynolds

**Affiliations:** Vashti, Va.


### A SUCCESSFUL CESAREAN SECTION IN COUNTRY PRACTICE.*

BY DR. S. A. REYNOLDS,

VASHTI, VA.

On February 14, 1905, I was called in consultation with Dr. E. S. Keen, to see Fannie Brown, a colored girl, thirteen years of age, who was then in labor at term and had been for twelve hours.

I made an examination and found the pelvic diameter less than before the Medical Society of Virginia, Norfolk, Va., October 24-27, 1905.

two inches, and as well as I could judge the diameters were only one and three-fourths inches.

After consultation we decided the only way of relief was to perform a Cesarean section.

We gave the patient a dose of morphia, left her in charge of a midwife, went to our homes, got our pocket-cases of instruments, and returned next morning to operate.

We made a second examination to see if we could not deliver her *per vaginam*, but found that utterly impossible, so we realized the seriousness of the situation, as neither of us had ever done a laparotomy.

We were poorly equipped for operating as we only had our pocket-cases of instruments, some carbolic acid and a few ligatures, an earthen pitcher and a bowl with a teakettle of hot water.

Our hospital was a log-cabin with one door, and a fireplace and a hole twelve inches square in the end of the house, without glass, for a window, our trained nurses were three old colored women, one of whom was a midwife, and our light a lamp without a chimney.

The day was very cold and the wind was high and blowing a perfect gale, so we had to keep both door and window closed.

We sterilized our instruments by washing them in carbolized water, and the silk and wire sutures in very hot carbolized water.

Our hands were washed with soap and water and rinsed in carbolized water.

With these equipments we began to operate at twelve o'clock, by Dr. Keen giving the chloroform, the patient having previously been prepared by placing her body across the bed with her feet resting on the floor, her clothing loosened and her body well washed with soap, and sponged off over the abdominal region and along line of proposed incision with carbolized water.

The patient took the chloroform beautifully and in five minutes was thoroughly under its influence, so at five minutes past twelve o'clock I began to operate by making an incision, by lamp light, along the median line from the umbilicus to the pubic arch, laying bare the uterus.

The abdominal walls pressed on the sides of the uterus, which served to prevent the blood from entering the abdominal cavity when the uterus was opened.

I next opened the uterus by making an incision from the fundus to near the cervix on the anterior side, just beneath the abdominal incision. I then promptly removed the child, placenta, clots, etc., from the uterine cavity. The uterus contracted rapidly, so we had some difficulty in putting in the suture.

I then put one suture in the middle of the uterine incision and removed all blood and blood-clots from the abdominal cavity.

We then promptly closed the abdominal incision with wire and silk sutures, sponged it off with carbolized water, dusted Tyree's antiseptic powder over the closed incision, dressed it with absorbent cotton which was held in place by strips of adhesive plaster, placed the patient in bed, completing the operation in thirty minutes. Just as we were ready to close the abdominal incision the patient began to give down under the anesthetic, which was promptly relieved by Dr. Keen catching hold of the tongue and drawing it forward. This delayed us several minutes as I had to hold back the intestines with both hands to prevent them from falling out of the abdominal cavity, for the patient was breathing hard and heavily.

In closing the abdominal incision, we only put in one row of sutures, carrying each suture completely through the abdominal wall including skin, muscular tissue and peritoneum. The patient stood the operation very well and was able to speak in a low tone in forty minutes from the time the chloroform was given.

I saw the patient the day following the operation ; reaction had set in ; she was doing well ; there was no fever and no pain except very little about the bladder when urinating, which was caused by using the catheter before the operation. She had been given a small quantity of milk and a little water. She had voided her urine without difficulty and was somewhat stronger than when we left her the day after the operation.

We called to see her on alternate days, so one of us saw her every day.

Dr. Keen called to see her the next day, the second after the operation, and finding her bowels distended and somewhat tympanitic, he gave her a teaspoonful of a saturated solution of Epsom salts every three hours till her bowels moved well.

I called the following day to see her and finding that her bowels had not moved, and as her stomach did not retain the salts I discontinued them, gave her a tablet of cascara comp., which gave her a few good actions from her bowels, and the tympanitic condition disappeared, which caused the patient to feel much better. The milk and soup diet was continued.

On the fourth day her temperature was $103\frac{3}{5}$; pulse 150, respiration 36 per minute. We gave her one five grain tablet of acetanalid every six hours and fifteen drops of mur. tinc. iron with 1-40 grain strychnine sulphate every six hours.

The acetanalid was discontinued when the temperature reached and remained at and below 100.

The iron and strychnine were continued until the patient recovered.

No change in diet.

On the sixth day the dressing was removed and, as the incision was healing nicely by primary union, it was sponged off with carbolized water, dusted with thymiodide and dressed with plain antiseptic gauze held in place by a strong bandage. The patient was given milk and soup for first two weeks and was then allowed to have some solid food. After ten days we dressed the abdominal wound every few days, washing the closed incision each time with carbolized water and using thymiodide and plain gauze as a dressing.

We removed the sutures in about three weeks, but kept the strong bandage around the abdomen for several weeks longer. The patient was permitted to sit up in four weeks and walk around in the room.

Under the above detailed treatment the patient got along nicely and had an uneventful recovery, was restored to perfect health and has been working in the field doing hard manual labor, and is now as strong and as vigorous as she was before the operation.

The only instruments used in this operation were a sharp-pointed and a probe-pointed bistoury and a needle with some silk and wire sutures. I made a small opening near the umbilicus with the sharp-pointed bistoury, large enough to admit my finger, and with it as a guide I made the balance of the incision with a probe-pointed bistoury.

The first point I desire to call your attention to is that our patient never suffered but very little pain, and that only when urinating or coughing, and that during her entire sickness no opiate or other anodyne was given her.

Second, That whilst the well-equipped city hospitals with their beautiful and attractive trained nurses which have been such a comfort and a blessing to suffering humanity, is the most suitable place for surgery, yet good and successful surgery can be done in the little log cabin in the country, with the many obstacles and disadvantages with which a country physician has to contend.

Third, That we should never be afraid to put forth our best effort to relieve our patients when it is absolutely demanded, however hazardous or difficult the task, with the means at our command.

